# Brief Notes on Pyorrhœa Alveolaris in India

**Published:** 1905-04

**Authors:** 


BRIEF NOTES ON PYORRHŒA ALVEOLARIS IN INDIA. By Major Andrew Buchanan.

In 1898 the writer observed that many prisoners in one of the large Indian jails were suffering from pyorrhœa. The disease had not been much noticed previously. On inquiry it was found that the disease was more prevalent in those districts where the famine had been most severe. There were two hundred cases in a population of about one thousand.

While it was difficult to get evidence as to its influence on the general health, it was reasonable to suppose that the crowding together of a large number of such cases would have an injurious effect. In a batch of ninety prisoners who had come from one of the districts most affected by the famine, over seventy were suffering from pyorrhœa. The pyorrhœa was cured, and in six weeks there had been an average gain in weight of eight pounds per man.

The improvement in the general condition of these men led one to believe that the curing of the pyorrhœa had considerable influence.

As spongy gums may arise from different causes, he has found the following classification useful:

1. Those due to scurvy, or some form of malnutrition.
2. Those due to mercurialism.
3. Those due to a lack of proper care (with these may be classed those that had scurvy, but in whom the gums had not returned to their normal condition).
4. Those due to loose teeth.

The majority of cases belong to group 3 and are almost all curable.

*Treatment.*—The tartar was removed and an antiseptic pushed into the pockets with a flat stick. Where the pockets were large they were slit up with a lancet, and powdered sulphate of copper was applied and rubbed into the gums for a few minutes. Where the cases were due to a slight scorbutic condition, antiscorbutic remedies were given at the same time.

It has been found advisable in the Indian jails to examine the gums of all prisoners on admission, remove all tartar and cure pyorrhœa, and then make the prisoners clean their teeth daily, so that if scurvy appears it can be readily detected, as the first signs are those which are seen in the gums.

